# Femoral Pulse Pressure Variation Is Not Interchangeable with Radial Pulse Pressure Variation during Living Donor Liver Transplantation

**DOI:** 10.3390/jpm12081352

**Published:** 2022-08-22

**Authors:** Doyeon Kim, Jin Hee Ahn, Sangbin Han, Justin Sangwook Ko, Mi Sook Gwak, Gaab Soo Kim

**Affiliations:** 1 Department of Anesthesiology and Pain Medicine, Inha University Hospital, Inha University School of Medicine, Incheon 22332, Korea; 2 Department of Anesthesiology and Pain Medicine, Kangbuk Samsung Hospital, Sungkyunkwan University School of Medicine, Seoul 03181, Korea; 3 Department of Anesthesiology and Pain Medicine, Samsung Medical Centre, Sungkyunkwan University School of Medicine, Seoul 06351, Korea

**Keywords:** cirrhosis, end-stage liver disease, hyperdynamic circulation, vascular resistance, vascular compliance, Bland–Altman plot, concordance, percentage error

## Abstract

The radial artery is commonly used as the site measuring pulse pressure variation (PPV) during surgery. Accurate measurement of circulating blood volume and timely interventions to maintain optimal circulating blood volume is important to deliver sufficient oxygen to tissues and organs. It has not rather than never studied in patients undergoing liver transplantation whether PPV measured at peripheral sites, such as the radial artery, do represent central PPV for evaluating blood volume. In this retrospective study, 51 liver transplant recipients were enrolled. The two PPVs had been automatically recorded every minute in electrical medical records. A total 1878 pairs of the two PPVs were collected. The interchangeability of PPV measured at the radial and the femoral artery was analyzed by using the Bland–Altman plot, four-quadrant plot, Cohen’s kappa (k), and receiver operating curve. The bias and limits of agreement of the two PPVs were −1.3% and −8.8% to 6.2%, respectively. The percentage error was 75%. The concordance rate was 65%. The Kappa of PPV-radial determining whether PPV-femoral was >13% or ≤13% was 0.64. We found that PPV-radial is not interchangeable with PPV-femoral during liver transplantation. Additionally, PPV-radial failed to reliably track changes of PPV-femoral. Lastly, the clinical decision regarding blood volume status (depletion or not) is significantly different between the two PPVs. Therefore, PPV-femoral may help maintain blood volume circulating to major organs including the newly transplanted liver graft for liver transplant recipients.

## 1. Introduction

The systemic circulation of cirrhotic patients undergoing liver transplantation, which is called hyperdynamic circulation, is different from other surgical populations, being featured by decreased splanchnic vascular resistance, increased vascular compliance, and increased plasma volume [1,2,3,4]. During liver transplantation surgery, total circulating volume fluctuates due to massive blood loss, rapid fluid infusion and transfusion, clamping/unclamping of major vessels, and changes in vascular resistance resulting from vasoactive mediators and drugs [5,6,7]. Thus, the accurate measurement of circulating blood volume and timely interventions to maintain optimal circulating blood volume is important to deliver sufficient oxygen to tissues and organs including newly transplanted liver.

Pulse pressure variation (PPV) is widely used as a dynamic index to evaluate intravascular circulating blood volume, since PPV has shown superiority in predicting fluid responsiveness compared to conventional static indices such as central venous pressure and pulmonary artery occlusion pressure [8,9,10,11]. The radial artery is commonly, instead of the femoral artery, used for measuring PPV. However, the peripheral artery does not represent central arterial pressure during liver transplantation, and the discrepancy worsens after the reperfusion phase [12]. This study suggests that PPV measured at the radial artery (PPV-radial) cannot represent PPV measured at the femoral artery (PPV-femoral) because PPV is derived by analyzing arterial pressure waveforms. Additionally, the arterial waveform is influenced by the arterial compliance that tends to change by greater degree at peripheral arteries such as the radial artery compared to central arteries such as the femoral artery [13].

Therefore, we hypothesized that PPV-radial is not interchangeable with PPV-femoral during liver transplantation. We investigated the agreement of PPV-radial and PPV-femoral by using the Bland–Altman plot with PPV-femoral being the reference value.

## 2. Materials and Methods

We conducted a chart review of patients who underwent elective adult-to-adult living donor liver transplantation in our institution between March 2018 and December 2018. We excluded patients if radial arterial catheterization or femoral arterial catheterization failed or if recipients had cardiac arrhythmias. Ethical approval for this retrospective observational study (IRB No. 2019-03-017-001) was provided by the Samsung medical center Institution Review Board, Seoul, Korea (Chairperson Prof Lee SG) on 13 March 2019, and informed consent was waived. This study was conducted according to the principles stated in the Helsinki Convention.

### 2.1. Anesthetic Management

Anesthetic management and hemodynamic monitoring were conducted under the standardized institutional protocol, as described elsewhere [3,14]. No premedication was administered. Mechanical ventilation was initiated with a tidal volume of 8 mL/predicted body weight, an inspired oxygen fraction of 0.5 using medical air at a gas flow rate of 2 L/min, and positive end expiratory pressure of 6 cm H_2_O. The frequency of ventilation was adjusted to maintain end-tidal CO_2_ of 35 to 40 mmHg. The depth of isoflurane was adjusted to maintain bispectral index value between 40 and 60. Large bore central venous catheter (Multi-Lumen Access Catheters, MAC™, Arrow international, Wayne, PA, USA) was inserted in the right internal jugular vein with a pulmonary arterial catheter (Swan-Ganz CCOmbo V, Edwards Lifesciences, Irvine, CA, USA). A 20-gauge catheter (Angiocath. IV catheters, fluorinated ethylene propylene polymer, 3.2 cm length, 0.7 mm internal diameter, 1.1 mm external diameter, NJ, BD) was inserted in the radial artery. A 20-gauge catheter (leader cath., arterial polyethylene catheter, 8.0 cm length, 0.6 mm internal diameter, 0.9 mm external diameter, Vygon, Ecouen, France) was inserted in the femoral artery and femoral vein, respectively, using the Seldinger technique [15]. Pressure transducers were placed at a height of 4/5 the anteroposterior diameter of the thoracic cage [12]. The zeroing was performed at ambient pressure. Crystalloids, synthetic colloids, albumins, and blood products were infused to maintain circulating blood volume. Transfusion was performed as described elsewhere [16]. To maintain mean arterial pressure (≥70 mmHg), vasoactive agents such as dopamine, norepinephrine, and vasopressin were administered as appropriate.

### 2.2. Acquisition of PPV Data

Pulse pressure (PP) was defined as the difference between systolic pressure and diastolic pressure within a single heartbeat. PPV was automatically calculated by the monitoring computer (Intellivue MP monitor, Philips Medical Systems, Best, The Netherlands; DX 2020 monitor, Dixtal, Sao Paulo, SP, Brazil) over a period of 8 s, and the values from 4 consecutive rounds were used to calculate the average value, which was the value shown on the monitor. PPV was calculated using the following formula: [17] PPV (%) = 100 (PPmax − PPmin)/[(PPmax + PPmin)/2]. PPV-femoral reflects central arterial pulse pressure variation and PPV-radial reflects peripheral arterial pulse pressure variation. The two PPV values were automatically recorded in the anesthesia electronic medical record every minute.

### 2.3. Statistical Analysis

Prior to the study, PPV-femoral and PPV-radial data in the overall phase of 5 patients were extracted and analyzed using the Bland–Altman analysis (α err = 0.05, power(1 − β err) = 0.8) for effect size calculation. The results were mean differences −1.23%, standard deviation of differences 1.82, assuming the maximum allowed difference between two values 5%, 1850 of pairs data were required. A total of 51 recipients’ PPV data were collected. The PPV-femoral, a value obtained from the central artery, was used as the reference value. The Bland–Altman plot was used to evaluate the agreement between PPV-femoral and PPV-radial [18]. The bias was calculated as the mean difference between PPV-femoral and PPV-radial. The limit of the agreement represents the range in which 95% of the differences between methods are expected to lie and was calculated as the bias ±1.96 standard deviation. The percentage error (%) was further calculated by dividing the 1.96 standard deviation by the mean of all measured PPV-femoral. Interchangeability was finally determined if the percentage error was <30% [19]. In addition, the concordance of the two PPVs was assessed using the four-quadrant plot [20]. The gap between the two consecutive PPV-femoral was the X-axis value, and the gap between the two consecutive PPV-radial was the Y-axis value. The concordance rate was calculated as the percentage of total number of values in the lower left or upper right quadrant of the four-quadrant plot. To refine the concordance analysis, data at the center of 15% of the mean of all analyzed PPVs were excluded from the measure of concordance rate. Trending ability was determined if the concordance rate was >92% [21]. Intravascular volume state was classified into depletion status or non-depletion status based on a PPV-femoral of 13% [22]. The Cohen’s kappa and receiver operating characteristic were used to determine if the volume status measured by PPV-radial agrees with the volume status measured by PPV-femoral. The area under the curve (AUC) was analyzed using Delong’s method [23]. In addition, sensitivity, specificity, and positive and negative predictive value of PPV-radial were also analyzed.

Continuous variables were expressed as mean (standard deviation) or median [interquartile range]. Categorical variables were expressed as frequency (%). Analyses were performed using SPSS 25 (SPSS Inc., Chicago, IL, USA) or MedCalc 19.4 (MedCalc Software, Ostend, Belgium).

## 3. Results

From the 51 patients (15 females and 36 males), 1878 pairs of the two PPVs were collected: 542 pairs during the dissection phase, 492 pairs during the anhepatic phase, and 841 pairs during the reperfusion phase. PPV-femoral ranged from 2 to 42%, while PPV-radial ranged from 2 to 39%. Demographic data are shown in Table 1. The indication for transplantation was viral origins including hepatitis B and C (*n* = 33), alcoholic cirrhosis (*n* = 8), and biliary cirrhosis (*n* = 6). The Model for End-stage Liver Disease score was 10 (8–19). There were no cases in which PPV could not be measured due to intraoperative arrhythmias or malfunction of the arterial catheters.

### 3.1. Agreement between PPV-Radial and PPV-Femoral

**Figure 1:**
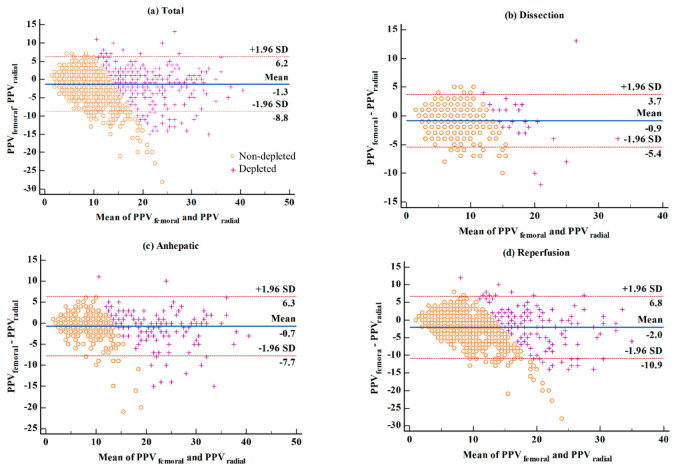
Bland–Altman plot evaluating the agreement of PPV-radial and PPV-femoral during. (**a**) Overall phases, (**b**) the dissection phase, (**c**) the anhepatic phase, and (**d**) the reperfusion phase. Based on value of PPV-femoral, non-depleted values (≤13%) are presented as orange circle (o), and depleted values (>13%) are presented as purple plus (+).

**Figure 2:**
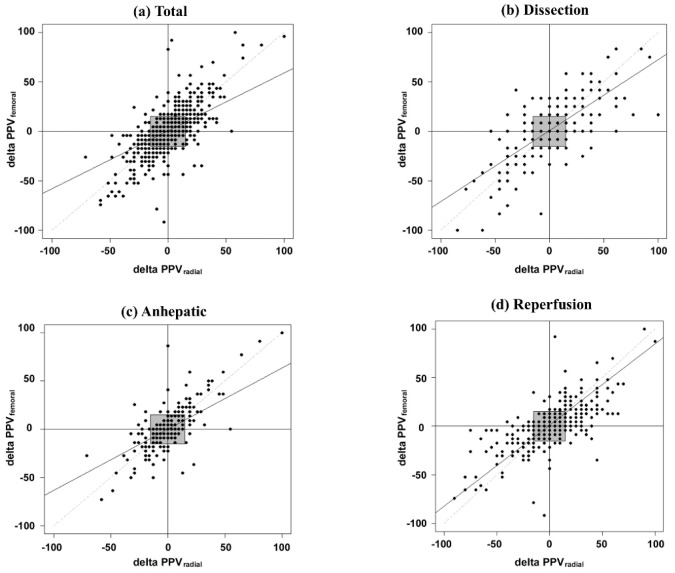
Four-quadrant plot to estimate the concordance rate between PPV-radial and PPV-femoral. The gray square is the central exclusion zone.

A Bland–Altman plot analyzing the agreement of PPV-radial and PPV-femoral is shown in Figure 1. PPV-radial tended to be greater than PPV-femoral and the bias of the two PPVs tended to increase as the surgery progressed by from the dissection phase to the reperfusion phase (overall, −1.3%; dissection, −0.9%; anhepatic, −0.7%; reperfusion, −2.0%). The limits of the agreement during the entire period (total), the dissection, the anhepatic, and the reperfusion were −8.8% to 6.2%, −5.2% to 3.9%, −7.7% to 6.3%, and −10.9% to 6.8%, respectively, while the percentage errors were 75.0%, 63.5%, 56.5% and 84.5%, respectively. As shown in Figure 2, the concordance rates during overall, dissection, anhepatic, and reperfusion phases were 65.0%, 86.7%, 89.5%, and 89.8%, respectively.

### 3.2. Agreement Regarding Volume Depletion between PPV-Radial and PPV-Femoral

Kappa to determine volume status (depletion or non-depletion) of PPV-radial and PPV-femoral was 0.64, 0.61, 0.77, and 0.51 during overall, dissection, anhepatic, and reperfusion phases (Table 2). The sensitivity of PPV-radial during overall, dissection, anhepatic, and reperfusion phases was 95.9%, 99.1%, 83.9%, and 49.9%, respectively. The specificity during overall, dissection, anhepatic, and reperfusion phases was 62.5%, 50.0%, 92.6%, and 95.6%, respectively (Table 3).

### 3.3. Analysis According to Intravascular Volume Status

**Figure 3:**
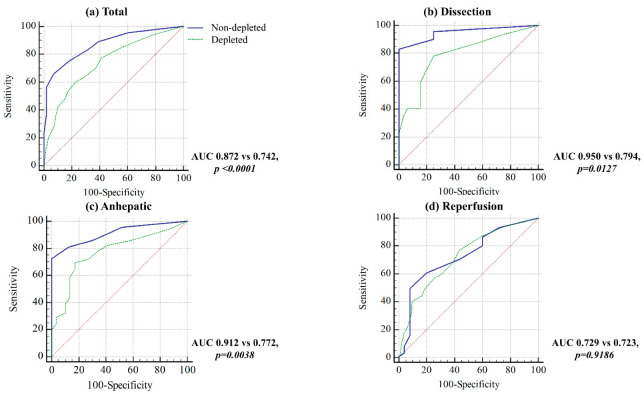
Receiver operating characteristics (ROC) curves of PPV-radial when intravascular volume was depleted (green dotted line) versus non-depleted (blue line). Red dotted line is reference line.

The agreement of the two PPVs according to the intravascular volume status is shown in Figure 3. The bias according to the volume status during each liver transplant phase was as follows: (non-depletion) overall, −1.5%; the dissection, −0.6%; the anhepatic, −0.5%; and the reperfusion, −2.5%; (depletion) overall, −1.1%; the dissection, −1.1%; the anhepatic, −0.6%; and the reperfusion, −1.6%; respectively. The limit of the agreement was as follows: (non-depletion) overall, −8.5% to 5.6%; the dissection, −4.7% to 3.5%; the anhepatic, −7.1% to 6.2%; and the reperfusion, −10.8% to 5.7%; (depletion) overall, −10.3% to 8.1%; the dissection, −9.7% to 7.5%; the anhepatic, −8.4% to 7.2%; and the reperfusion, −12.2% to 9.1%; respectively. The percentage error was as follows: (non-depletion) overall, 94.6%; the dissection, 65.0%; the anhepatic, 88.1%; and the reperfusion, 101.8%; (depletion) overall, 47.9%; the dissection, 49.0%; the anhepatic, 38.0%; and the reperfusion, 56.8%; respectively. The AUC of PPV-radial during the non-depletion status was significantly greater than the AUC of PPV-radial during the depletion status in overall phases (0.87 vs. 0.74 (*p* < 0.001)), the dissection (0.95 vs. 0.79 (*p* = 0.013)), and the anhepatic (0.91 vs. 0.77 (*p* = 0.004)), whereas there was no significant difference during the reperfusion (0.73 versus 0.72 (*p* = 0.919)) (Figure 3).

## 4. Discussion

In the current study, we determined inconsistent agreement between PPV-radial and PPV-femoral for cirrhotic patients undergoing liver transplantation. Although previous studies demonstrated the differences in arterial blood pressure and pulse pressure between the central and the peripheral during liver transplantation, there have been no studies determining the difference in arterial PPV. Our data demonstrated that PPV-radial does not reflect the central intravascular volume status during liver transplantation well. All analyzed statistical parameters including agreement, concordance, sensitivity, specificity, Kappa and AUC suggested that PPV-radial was not interchangeable with PPV-femoral during any surgical phases of liver transplantation. The poor interchangeability was maintained even when the circulating volume was sufficient although diagnostic value of PPV-radial was relatively greater when the circulating volume was sufficient then when the circulating volume was depleted. These data also suggested that intravascular circulating volume status of peripheral is considerable different from that of central, being in consistent with previous research demonstrating significantly different systemic vascular resistance between the gut, the central, and the peripheral [2,4,24]. Overall, it could be concluded that peripheral PPV is not interchangeable with central PPV irrespective of circulating volume status or surgical phase; thus, PPV-femoral should be used to accurately evaluate intravascular circulating volume status of central and to maintain adequate oxygen delivery to major organs including the newly transplanted liver graft.

In the current study, there was a disagreement between PPV-radial and PPV-femoral, and PPV-radial was insufficient in aspects of evaluating the adequacy of intravascular blood volume circulating in central body parts in clinical practice. Previous studies reported that peripheral arterial pressure does not reflect central arterial pressure during liver transplantation [25,26]. In addition, central arterial pulse pressure is also known to be different from peripheral pulse pressure: peripheral arterial waveform generally shows higher systolic pressure and lower diastolic pressure with greater pulse pressure compared to central pulse pressure [27]. The difference may be attributable to the difference in vessel composition: central arteries are rich in elastic fibers, peripheral arteries are rich in smooth muscle cells, and arterial compliance gradually decreases toward the peripheral [28]. Additionally, vasodilators such as nitric oxide are known to dominantly act on the vascular smooth muscle [29,30]. Liver transplant recipients with end-stage liver disease who experience significant changes in vascular resistance and vascular compliance may have greater difference in arterial resistance and arterial compliance between the central and peripheral [24,31], resulting in greater difference in arterial blood pressure, waveform, pulse pressure, and pulse pressure variation [16]. Therefore, fluid management using PPV-femoral monitoring, which reflects central volume status, is required during liver transplantation because it is relatively less affected by vasoactive drugs or surgical manipulation.

There are several limitations in the current study. First, we collected and analyzed the data retrospectively. For LT surgery in our institution, anesthesia and surgical methods were standardized by an LT specialist surgeon and anesthesiologist, and all PPV values were automatically collected in the anesthesia electronic medical record every minute. Therefore, our results are derived from well-controlled data and are not different from the data quality of a prospective study. Second, we did not include patients who underwent deceased donor liver transplantation. Recipients undergoing deceased donor liver transplantation generally show greater hemodynamic or circulatory deteriorations and intraoperative fluctuations. Thus, it could be deduced that the difference in intravascular circulating volume or PPV between the central and the peripheral are greater compared to living donor liver transplantation. Third, the catheters inserted into the femoral artery and the radial artery were different with different characteristics including material, length, internal diameter, and thickness. The pulse pressure variation is a value that reflects the relative variation according to the ventilation cycle at each site and is considered to be more affected by the characteristics of the blood vessel itself than the type of catheter.

In conclusion, PPV-radial was not interchangeable with PPV-femoral during living donor liver transplantation, irrespective of the amount of intravascular circulating volume and surgical phases. Therefore, PPV-femoral monitoring is highly recommended for accurately estimating intravascular blood volume circulating the central body parts and major organs including the newly transplanted liver graft.

## Figures and Tables

**Table 1:** Patients characteristics.

Variables	Descriptive Statistics
**Donor factor**	
Gender (male, %)	36 (71)
Age (years)	33 [25–48]
Body mass index (kg/m2)	24 [21–26]
Graft-to-recipient body weight ratio	1.06 [0.87–1.20]
Macrosteatosis	15 (29)
**Recipient factor**	
Gender (male)	38 (75)
Age (years)	57 [51–62]
Body mass index (kg/m2)	24.3 [21.7–27.2]
MELD score	10 (8–19)
Etiology	
Viral	33 (65)
Alcoholic	8 (16)
Biliary	6 (12)
Cryptogenic	4 (8)
Preoperative laboratory findings	
White blood cell (×103/μL)	4.06 [3.02–5.02]
Hemoglobin (g/dL)	12.0 [9.7–13.6]
Hematocrit (%)	35.3 [28.7–40.7]
Platelet (×103/μL)	100 [51.0–122.0]
Prothrombin time (INR)	1.27 [1.07–1.62]
Albumin (g/dL)	3.4 [2.9–4.2]
Total bilirubin (mg/dL)	1.6 [0.7–5.3]
Creatinine (mg/dL)	0.75 [0.63–0.96]
Glucose (mg/dL)	115 [90–173]
Sodium (mmol/L)	138 [134–140]
**Intraoperative**	
Total anesthesia time (min)	494 [465–559]
Operation time (min)	401 [363–460]
Cold ischemia time (min)	73 [63–84]
Warm ischemia time (min)	37 [34–47]
Fluid administration and transfusion	
Crystaloid (mL)	4850 [4050–6400]
5% albumin (mL)	890 [760–1100]
Synthetic colloid (mL)	1000 [500–1000]
Red blood cell (unit)	0 (0–2)
Fresh frozen plasma (unit)	0 (0–2)
Single donor platelet (unit)	0 (0–0)
Cryoprecipitate (unit)	0 (0–0)

Data are expressed as median [interquartile range] or frequency (%). MELD: model of end-stage liver disease.

**Table 2:** Agreement regarding intravascular volume depletion between PPV-radial and PPV-femoral.

Phase	k, Kappa (95%CI)	Agreement, *n* (%)	Disagreement, *n* (%)
Overall	0.641 (0.614, 0.669)		
Non-depleted		1312 (70)	56 (3)
Depleted		319 (17)	191 (10)
Dissection	0.607 (0.491, 0.722)		
Non-depleted		474 (87)	4 (1)
Depleted		32 (6)	32 (6)
Anhepatic	0.771 (0.712, 0.830)		
Non-depleted		289 (59)	23 (5)
Depleted		151 (31)	29 (6)
Reperfusion	0.510 (0.447, 0.572)		
Non-depleted		539 (64)	25 (3)
Depleted		138 (16)	139 (17)

**Table 3:** Evaluation of diagnostic power of PPV-radial.

Phase	Sensitivity	Specificity	Accuracy	Positive Predictive Value	Negative Predictive Value
Overall	85	87	87	63	95
Dissection	89	93	93	50	99
Anhepatic	87	91	89	84	93
Reperfusion	85	80	81	50	96

Data are expressed as %.

## Data Availability

All data generated or analyzed during this study are included in this published article.

## References

[1] Lee R.F., Glenn T.K., Lee S.S. (2007). Cardiac dysfunction in cirrhosis. Best Pract. Res. Clin. Gastroenterol..

[2] Moller S., Henriksen J.H. (2008). Cardiovascular complications of cirrhosis. Gut.

[3] Han S., Lee J.H., Kim G., Ko J.S., Choi S.J., Kwon J.H., Heo B.Y., Gwak M.S. (2015). Bioreactance Is Not Interchangeable with Thermodilution for Measuring Cardiac Output during Adult Liver Transplantation. PLoS ONE.

[4] Newby D.E., Hayes P.C. (2002). Hyperdynamic circulation in liver cirrhosis: Not peripheral vasodilatation but ‘splanchnic steal’. QJM Int. J. Med..

[5] Krenn C.G., Hoda R., Nikolic A., Greher M., Chevtchik O.O., Steltzer H. (2004). Assessment of ventricular contractile function during orthotopic liver transplantation. Transpl. Int..

[6] Arranz J., Soriano A., Garcia I., Concepción M.T., Navarro J., Artega A., Fiella X., Bravi P., Barrera M., Escribano S. (2003). Effect of proinflammatory cytokines (IL-6, TNF-alpha, IL-1beta) on hemodynamic performance during orthotopic liver transplantation. Transplant. Proc..

[7] Aggarwal S., Kang Y., Freeman J.A., Fortunato F.L., Pinsky M.R. (1993). Postreperfusion syndrome: Hypotension after reperfusion of the transplanted liver. J. Crit. Care.

[8] Cole R. (2008). Does Central Venous Pressure Predict Fluid Responsiveness?. Chest.

[9] Marik P.E., Baram M., Vahid B. (2008). Does Central Venous Pressure Predict Fluid Responsiveness?*: A Systematic Review of the Literature and the Tale of Seven Mares. Chest.

[10] Marik P.E., Cavallazzi R. (2013). Does the Central Venous Pressure Predict Fluid Responsiveness? An Updated Meta-Analysis and a Plea for Some Common Sense. Crit. Care Med..

[11] Huber W., Umgelter A., Reindl W., Franzen M., Schmidt C., von Delius S., Geisler F., Eckel F., Fritsch R., Siveke J. (2008). Volume assessment in patients with necrotizing pancreatitis: A comparison of intrathoracic blood volume index, central venous pressure, and hematocrit, and their correlation to cardiac index and extravascular lung water index. Crit. Care Med..

[12] Arnal D., Garutti I., Perez-Peña J., Olmedilla L., Tzenkov I.G. (2005). Radial to femoral arterial blood pressure differences during liver transplantation. Anaesthesia.

[13] Mayer J., Boldt J., Poland R., Peterson A., Manecke G.R. (2009). Continuous Arterial Pressure Waveform–Based Cardiac Output Using the FloTrac/Vigileo: A Review and Meta-analysis. J. Cardiothorac. Vasc. Anesth..

[14] Han S., Park H.-W., Song J.H., Gwak M.S., Lee W.J., Kim G., Lee S.-K., Ko J.S. (2016). Association Between Intraoperative Platelet Transfusion and Early Graft Regeneration in Living Donor Liver Transplantation. Ann. Surg..

[15] Song I.-K., Kim E.-H., Lee J.-H., Jang Y.-E., Kim H.-S., Kim J.-T. (2018). Seldinger vs. modified Seldinger techniques for ultrasound-guided central venous catheterisation in neonates: A randomised controlled trial. Br. J. Anaesth..

[16] Han S., Kwon J.H., Jung S.H., Seo J.Y., Jo Y.J., Jang J.S., Yeon S.M., Jung S.H., Ko J.S., Gwak M.S. (2018). Perioperative Fresh Red Blood Cell Transfusion May Negatively Affect Recipient Survival After Liver Transplantation. Ann. Surg..

[17] Michard F., Chemla D., Richard C., Wysocki M., Pinsky M.R., Lecarpentier Y., Teboul J.-L. (1999). Clinical Use of Respiratory Changes in Arterial Pulse Pressure to Monitor the Hemodynamic Effects of PEEP. Am. J. Respir. Crit. Care Med..

[18] Bland J.M., Altman D.G. (1986). Statistical methods for assessing agreement between two methods of clinical measurement. Lancet.

[19] Critchley L.A.H., Critchley J.A.J.H. (1999). A Meta-Analysis of Studies Using Bias and Precision Statistics to Compare Cardiac Output Measurement Techniques. J. Clin. Monit. Comput..

[20] Saugel B., Grothe O., Wagner J.Y. (2015). Tracking Changes in Cardiac Output: Statistical considerations on the 4-quadrant plot and the polar plot methodology. Anesth. Analg..

[21] Critchley L.A., Lee A., Ho A.M.-H. (2010). A Critical Review of the Ability of Continuous Cardiac Output Monitors to Measure Trends in Cardiac Output. Anesth. Analg..

[22] Hadian M., Severyn D.A., Pinsky M.R. (2011). The effects of vasoactive drugs on pulse pressure and stroke volume variation in postoperative ventilated patients. J. Crit. Care.

[23] Delong E.R., Delong D.M., Clarke-Pearson D.L. (1988). Comparing the Areas under Two or More Correlated Receiver Operating Characteristic Curves: A Nonparametric Approach. Biometrics.

[24] Mutchler S.M., Straub A.C. (2015). Compartmentalized nitric oxide signaling in the resistance vasculature. Nitric Oxide.

[25] Lee M., Weinberg L., Pearce B., Scurrah N., Story D., Pillai P., McCall P.R., McNicol L.P., Peyton P.J. (2015). Agreement between radial and femoral arterial blood pressure measurements during orthotopic liver transplantation. Crit. Care Resusc. J. Australas. Acad. Crit. Care Med..

[26] Bouchard-Dechêne V., Couture P., Su A., Deschamps A., Lamarche Y., Desjardins G., Levesque S., Denault A.Y. (2018). Risk Factors for Radial-to-Femoral Artery Pressure Gradient in Patients Undergoing Cardiac Surgery with Cardiopulmonary Bypass. J. Cardiothorac. Vasc. Anesth..

[27] Remington J.W. (1960). Contour changes of the aortic pulse during propagation. Am. J. Physiol. Leg. Content.

[28] Kanazawa M., Fukuyama H., Kinefuchi Y., Takiguchi M., Suzuki T. (2003). Relationship between Aortic-to-radial Arterial Pressure Gradient after Cardiopulmonary Bypass and Changes in Arterial Elasticity. Anesthesiology.

[29] Iwakiri Y., Groszmann R.J. (2006). The hyperdynamic circulation of chronic liver diseases: From the patient to the molecule. Hepatology.

[30] Ignarro L.J., Byrns R.E., Wood K.S. (1987). Endothelium-dependent modulation of cGMP levels and intrinsic smooth muscle tone in isolated bovine intrapulmonary artery and vein. Circ. Res..

[31] Dart A.M., Kingwell A.B. (2001). Pulse pressure—A review of mechanisms and clinical relevance. J. Am. Coll. Cardiol..

